# Prospective Evaluation of Low-Dose Theophylline as an Add-On Therapy in Patients With Chronic Obstructive Pulmonary Disease (COPD)

**DOI:** 10.7759/cureus.94207

**Published:** 2025-10-09

**Authors:** Sumaira Malik, Ateka Ikram, Abdullah Elrefae, Sohail Khan Raja, Maryam Atta, Muhammad Rizwan Umer, Muhammad Iftikhar Khattak, Kashaf Malik

**Affiliations:** 1 Department of Internal Medicine, Russells Hall Hospital, Dudley, GBR; 2 Department of Respiratory, Basildon University Hospital, Basildon, GBR; 3 Department of Trauma and Orthopaedics, AlBashir Hospital, Amman, JOR; 4 Department of Pulmonology, Abbas Institute of Medical Sciences, Muzaffarabad, PAK; 5 Department of Medicine, Azad Jammu and Kashmir Medical College, Muzaffarabad, PAK; 6 Department of Trauma and Emergency, Royal Sussex County Hospital, Brighton and Hove, GBR; 7 Department of Research and Development, Celestial and Dimanche, Muzaffarabad, PAK; 8 Department of Medicine, Rawal Institute of Health Sciences, Islamabad, PAK

**Keywords:** adjunct therapy, copd, exacerbation, low-dose theophylline, observational study, pakistan, spirometry

## Abstract

Background: Chronic obstructive pulmonary disease (COPD) is still a big health concern across the globe, and better pharmacological techniques are needed to better control the condition.

Objective: This study aimed to find out whether low-dose theophylline may help COPD patients get better when used with other treatments.

Methodology: This prospective observational research was place at the Department of Pulmonology of Abbas Institute of Medical Sciences (AIMS) in Muzaffarabad, Pakistan, from April 2023 to March 2024. A total of 132 individuals with stable moderate to severe COPD who were at least 40 years old were recruited and given low-dose theophylline (300 mg once a day) along with their normal inhaled treatment. We checked up on the patients every three months for a year. The main results were spirometric measures (FEV₁, FVC, FEV₁/FVC), COPD Assessment Test (CAT) scores, Modified Medical Research Council (mMRC) dyspnea grades, how often exacerbations happened, how well people followed their treatment, and any side effects. We used IBM SPSS Statistics for Windows, Version 26.0 (Released 2019; IBM Corp., Armonk, New York, United States), to look at the data and ran repeated measures ANOVA and chi-squared testing.

Results: The mean FEV₁ increased from 1.12±0.25 L at baseline to 1.33±0.28 L at 12 months (p<0.001) and FVC increased from 2.01±0.42 L to 2.20±0.44 L (p<0.01). CAT scores dropped from 22.8 to 15.6 and mMRC grades from 2.92 to 2.02 (both p<0.001). A total of 59 patients (44.7%) experienced no exacerbations during follow-up. Therapy adherence >80% was achieved in 117 patients (88.6%), and 94 patients (71.2%) reported no adverse effects.

Conclusion: Low-dose theophylline may offer a well-tolerated, cost-effective adjunct to conventional COPD treatment in resource-limited settings.

## Introduction

Chronic obstructive pulmonary disease (COPD) is a chronic respiratory disorder characterized by persistent airway inflammation and progressive airflow limitation. It is primarily caused by long-term exposure to harmful particles or gases, with cigarette smoke being the most common risk factor [1,2]. COPD remains one of the leading causes of morbidity and mortality worldwide, contributing to over three million deaths annually and imposing a significant burden on healthcare systems and patients' quality of life [3].

Despite advances in pharmacotherapy, mainly through long-acting bronchodilators, inhaled corticosteroids (ICS), and combination inhalers, many patients continue to experience symptom worsening and acute exacerbations, indicating an unmet need for additional treatment options [4]. Theophylline, a methylxanthine derivative with bronchodilator and anti-inflammatory effects, was widely prescribed in the past for airway diseases [5]. However, its use has markedly declined because of its narrow therapeutic index, pharmacokinetic variability, and systemic adverse effects at higher doses [6].

Early mechanistic and clinical studies suggested that low-dose theophylline could restore corticosteroid responsiveness by enhancing histone deacetylase-2 (HDAC2) activity, modulate neutrophilic inflammation, and improve mucociliary clearance [7,8]. These findings generated interest in its potential role as an adjunctive therapy, particularly in resource-limited settings where modern inhaled therapies may be less available [9].

Nevertheless, contemporary high-quality evidence does not support the efficacy of low-dose theophylline in COPD. In the TWICS randomized trial involving more than 1,500 patients, adding low-dose theophylline to ICS did not reduce the rate of moderate or severe exacerbations compared to placebo [10]. Similarly, the TASCS trial found no benefit of low-dose theophylline, with or without low-dose prednisone, in reducing exacerbation rates or improving lung function and quality of life [11]. A systematic review and meta-analysis further reported that low-dose theophylline added to ICS did not reduce exacerbation frequency and, in fact, increased hospitalization and mortality risks [12].

Moreover, the narrow therapeutic window of theophylline remains a clinical concern, as case reports have described acute-on-chronic toxicity in long-term users, presenting with nonspecific symptoms, seizures, and respiratory failure [13,14], highlighting the risks even at so-called "therapeutic" levels without regular monitoring. Although recent randomized controlled trials (RCTs) such as TWICS and TASCS did not demonstrate significant reductions in exacerbations, these trials were conducted predominantly in high-income settings and may not reflect the clinical and socioeconomic realities of COPD management in Pakistan, where access to inhaled therapies is often limited. Therefore, pragmatic observational studies are needed to generate locally relevant evidence, particularly because low-dose theophylline may confer anti-inflammatory and corticosteroid-sensitizing effects in real-world populations. Current evidence suggests that low-dose theophylline offers limited clinical benefit while retaining safety concerns; accordingly, the objective of this study was to evaluate whether low-dose theophylline can improve outcomes in COPD patients when used as an adjunct to standard therapy.

## Materials and methods

Study design and setting

This was a prospective observational study conducted at the Department of Pulmonology of Abbas Institute of Medical Sciences (AIMS) in Muzaffarabad, Pakistan, over a 12-month period from April 2023 to March 2024. The observational design was chosen because it allows the evaluation of the real-world effectiveness and tolerability of low-dose theophylline as an adjunct to standard COPD management in a representative patient population. RCTs, while considered the gold standard, often exclude patients with significant comorbidities or socioeconomic barriers, limiting generalizability [1,2]. In contrast, an observational approach captures outcomes in patients typically seen in routine practice, which is especially important in low-resource settings like Pakistan, where COPD prevalence and treatment challenges remain high [3,4]. Furthermore, COPD is a heterogeneous disease with complex pathophysiology driven largely by chronic exposure to cigarette smoke and airway inflammation [5,6], making it vital to study therapeutic responses outside the restrictive framework of RCTs.

Another important rationale for this design was feasibility and ethics. In a resource-limited setting, conducting a placebo-controlled RCT would have required extensive funding, larger sample sizes, and potentially withholding beneficial therapies, raising ethical concerns. Instead, our design ensured all patients continued their standard inhaled therapy, while low-dose theophylline was introduced as an add-on based on prior evidence of its potential to enhance corticosteroid responsiveness and provide anti-inflammatory benefits [8,10,11]. Although some recent RCTs (Devereux et al. [8,10]; Jenkins et al. [11]) did not confirm significant reductions in exacerbation rates, they highlighted the need for further pragmatic studies to clarify its role [9,10]. Additionally, reports from regional studies, including Ramsdell et al., in San Diego [15], Farooq et al., in Pakistan [16], and Haddad et al., in Jordan [17], as well as case experiences on theophylline toxicity published in Cureus [13,14], emphasize both the therapeutic potential and safety challenges associated with this drug in real-world populations. By situating our study in this context, we aimed to generate locally relevant, practice-oriented evidence that complements global literature and informs COPD management strategies in similar settings.

Inclusion and exclusion criteria

Patients were eligible if they were 40 years or older with a confirmed diagnosis of stable moderate to severe COPD based on the Global Initiative for Chronic Obstructive Lung Disease (GOLD) guidelines [18]. This age threshold was chosen because COPD prevalence and severity increase significantly after the fourth decade of life, while younger patients (<40 years) may have asthma or other differential diagnoses that could confound outcomes [1-3,6,15]. Eligible patients were required to be on standard maintenance inhaled therapy (bronchodilators with or without corticosteroids), provide informed consent, and demonstrate adherence to follow-up visits and treatment. Patients were excluded if they had a recent COPD exacerbation or hospitalization, theophylline hypersensitivity, other chronic lung diseases (e.g., asthma, tuberculosis, or bronchiectasis), or significant hepatic or renal impairment, were pregnant or breastfeeding, or used methylxanthine within the prior six months. These criteria ensured a homogeneous COPD cohort, minimized confounding, and were consistent with prior regional and international studies on low-dose theophylline [3-5,7,8,11,12,16,17,19,20-23].

Sampling and sample size

A total of 148 patients were initially enrolled using convenience sampling from the outpatient and inpatient COPD population at the Department of Pulmonology of AIMS, Muzaffarabad. This approach was chosen due to the single-center design and practical constraints, with the aim of recruiting all consecutive patients who met the inclusion criteria during the 12-month study period. Although convenience sampling was used, the study cohort reflects typical COPD patients in a tertiary care hospital, enhancing external validity. Convenience sampling is widely used in exploratory observational studies, particularly in low- and middle-income settings, to reflect real-world patient characteristics and ensure feasibility.

During the follow-up, 16 patients were lost due to withdrawal of consent, poor follow-up adherence, or unrelated health issues, leaving 132 patients who completed the study. Although a formal a priori sample size calculation was not performed, the adequacy of the final sample was supported through a post hoc power analysis. Based on the observed improvement in mean FEV₁ over 12 months (effect size d≈0.35), the available sample of 132 patients provided >80% power at a significance level of 0.05, confirming sufficient statistical precision to detect clinically meaningful differences.

For transparency, the general formula for calculating sample size for comparing means is as follows: $n = \frac{(Z_{\alpha/2} + Z_{\beta})^2 \cdot 2\sigma^2}{\Delta^2}$. Here, n is the required sample size per group; Z_α/2_ is the standard normal deviate for type I error (e.g., 1.96 for 95% CI); Z_β_ is the standard normal deviate for type II error (e.g., 0.84 for 80% power); σ^2^ is the variance of outcome measure (e.g., FEV₁); and Δ is the expected mean difference between groups.

Applying this retrospectively, our achieved sample size fell within the range reported in other observational studies of low-dose theophylline in COPD, including Farooq et al. [16] and Haddad et al. [17]. Thus, while the absence of a prospective calculation is a limitation, the study retained adequate statistical power and external comparability.

Data collection

Data were collected using a standardized clinical proforma routinely employed in the Department of Pulmonology of AIMS (attached in the Appendices). Baseline and follow-up assessments were conducted at months 3, 6, 9, and 12. Collected variables included demographics, smoking history, spirometry (FEV₁, FVC, FEV₁/FVC), COPD Assessment Test (CAT) scores [10], and Modified Medical Research Council (mMRC) dyspnea grades [11]. Each participant received 300 mg of low-dose theophylline once daily in addition to their regular inhaled maintenance regimen, consistent with earlier studies demonstrating potential anti-inflammatory and steroid-sparing benefits of low-dose theophylline [12-14]. Follow-up evaluations assessed symptom burden, lung function, exacerbation frequency, adherence, and adverse events. Monitoring for safety was prioritized given reports of dose-related theophylline toxicity, as highlighted in previous reports [3-5,7,8,11,12,16,17,21,23], ensuring that both efficacy and risk were systematically captured.

Statistical analysis

Data were analyzed using IBM SPSS Statistics for Windows, Version 26.0 (Released 2019; IBM Corp., Armonk, New York, United States). Continuous variables were expressed as means±standard deviations and categorical data as proportions. Longitudinal changes in lung function (FEV₁, FVC, FEV₁/FVC) and symptom scores (CAT, mMRC) were analyzed using repeated measures ANOVA, while post hoc Bonferroni tests identified pairwise differences across follow-up intervals. Exacerbation frequencies were compared using the chi-squared test. A two-tailed p<0.05 was considered statistically significant. The chosen methods align with prior pharmacotherapy studies in COPD evaluating longitudinal outcomes [3-5,7,8,11,12,16,17-19,21,23]. Additionally, a post hoc power calculation confirmed sufficient power for the observed sample size, reinforcing statistical robustness despite the absence of an a priori calculation.

Ethics approval and consent to participate

The study was approved by the Ethical Review Board of Abbas Institute of Medical Sciences (approval number: 1181/AIMS/2023). Written informed consent was obtained from all participants prior to enrollment, in accordance with the Declaration of Helsinki.

## Results

Out of the 132 patients analyzed, the majority were in the 50-59-year age group (n=46; 34.85%), followed by 60-69 years (n=39; 29.55%), 40-49 years (n=28; 21.21%), and ≥70 years (n=19; 14.39%), as shown in Table 1. Males accounted for 65.91% (n=87) and females 34.09% (n=45). Regarding smoking status, 38.64% (n=51) were current smokers, 44.7% (n=59) were ex-smokers, and 16.67% (n=22) had never smoked. In terms of COPD severity based on GOLD staging, most patients were in stage III (severe) at 50% (n=66), followed by stage II (moderate) at 34.85% (n=46) and stage IV (very severe) at 15.15% (n=20).

**Table 1:** Baseline demographic and clinical characteristics (n=132) COPD: chronic obstructive pulmonary disease; GOLD: Global Initiative for Chronic Obstructive Lung Disease

Category	Variable	Frequency (n)	Percentage (%)
Age group (years)	40-49	28	21.21%
	50-59	46	34.85%
	60-69	39	29.55%
	≥70	19	14.39%
Gender	Male	87	65.91%
	Female	45	34.09%
Smoking status	Current smoker	51	38.64%
	Ex-smoker	59	44.7%
	Never smoker	22	16.67%
COPD severity (GOLD)	Stage II (moderate)	46	34.85%
	Stage III (severe)	66	50%
	Stage IV (very severe)	20	15.15%

Spirometric outcomes demonstrated progressive improvement across the 12-month follow-up. The mean FEV₁ increased from 1.12±0.25 L at baseline to 1.33±0.28 L (F=12.87; df=4,520; p<0.001), while the mean FVC rose from 2.01±0.42 L to 2.20±0.44 L (F=4.92; df=4,520; p<0.01). The FEV₁/FVC ratio also improved from 55.72±5.11% to 60.45±5.12% (F=6.15; df=4,520; p<0.01), indicating statistically significant gains in lung function with low-dose theophylline therapy (Table 2).

**Table 2:** Mean spirometric values over time (n=132)

Time point		FEV₁ (L) mean±SD (95% CI)	FVC (L) mean±SD (95% CI)	FEV₁/FVC ratio % mean±SD (95% CI)
Baseline		1.12±0.25 (1.08-1.16)	2.01±0.42 (1.94-2.08)	55.72±5.11 (54.84-56.60)
3 months		1.20±0.26 (1.16-1.25)	2.08±0.43 (2.01-2.15)	57.42±5.09 (56.54-58.30)
6 months		1.24±0.29 (1.19-1.30)	2.12±0.45 (2.05-2.20)	58.33±5.17 (57.44-59.22)
9 months		1.28±0.27 (1.23-1.33)	2.15±0.41 (2.08-2.22)	59.53±5.08 (58.65-60.41)
12 months		1.33±0.28 (1.28-1.38)	2.20±0.44 (2.13-2.27)	60.45±5.12 (59.56-61.34)
ANOVA	F	12.87	4.92	6.15
	df	4,520	4,520	4,520
	p	p<0.01	p<0.01	p<0.01

Symptom burden decreased substantially during the study period. The mean CAT score fell from 22.8±3.6 at baseline to 15.6±2.9 at 12 months (F=25.33; df=4,520; p<0.001), and the mean mMRC dyspnea grade declined from 2.92±0.61 to 2.02±0.49 (F=19.72; df=4,520; p<0.001). These reductions reflect significant clinical improvement in health status and dyspnea severity following add-on theophylline therapy (Table 3).

**Table 3:** Changes in symptom scores over 12 months (n=132) CAT: Chronic Obstructive Pulmonary Disease Assessment Test; mMRC: Modified Medical Research Council

Time point		CAT score mean±SD (95% CI)	mMRC dyspnea grade mean±SD (95% CI)
Baseline		22.8±3.6 (22.2-23.4)	2.92±0.61 (2.81-3.02)
3 months		20.4±3.3 (19.8-21.0)	2.64±0.59 (2.54-2.74)
6 months		18.5±3.1 (17.9-19.1)	2.41±0.56 (2.31-2.50)
9 months		17.1±3.0 (16.5-17.7)	2.20±0.50 (2.11-2.29)
12 months		15.6±2.9 (15.1-16.2)	2.02±0.49 (1.93-2.11)
Net change		−7.2	−0.9
ANOVA	F	25.33	19.72
	df	4,520	4,520
	p	p<0.01	p<0.001

During the 12-month follow-up, 44.7% (n=59) of patients experienced no exacerbations, 28.79% (n=38) had one, 15.91% (n=21) had two, and 10.61% (n=14) experienced three or more exacerbations (Table 4). The mean number of exacerbations per patient was 1.12±0.81. The distribution was statistically significant (p<0.001; χ²=36.55; df=3), suggesting that low-dose theophylline was effective in reducing exacerbation frequency.

**Table 4:** Frequency of COPD exacerbations during follow-up (n=132) χ²=36.55; df=3 COPD: chronic obstructive pulmonary disease

Exacerbation frequency	Number of patients	Percentage (%)	P-value
0	59	44.7%	<0.001
1	38	28.79%	
2	21	15.91%	
≥3	14	10.61%	
Mean exacerbations/patient	Mean±SD	1.12±0.81	

Therapy adherence was high, with 88.64% (n=117) of patients maintaining >80% adherence (Table 5). Adverse events were reported in 28.79% (n=38) of patients, the most common being mild gastrointestinal upset (n=19; 14.39%) and mild headache (n=11; 8.33%). Only 2.27% (n=3) discontinued treatment due to side effects. Notably, 71.21% (n=94) of patients reported no adverse events, suggesting that low-dose theophylline was generally well-tolerated.

**Table 5:** Adherence and adverse events (n=132) Some patients experienced more than one adverse symptom, so the total number of symptom reports exceeds the number of individuals with adverse events (n=38).

Category	Variable	Frequency (n)	Percentage (%)
Therapy adherence	Therapy adherence (>80%)	117	88.64%
	Treatment discontinuation due to side effects	3	2.27%
Adverse events	Any adverse event reported (one or more)	38	28.79%
	Mild gastrointestinal upset	19	14.39%
	Mild headache	11	8.33%
	No adverse events reported	94	71.21%

## Discussion

This prospective observational study found that adjunctive low-dose theophylline in COPD management over 12 months led to significant improvements in lung function, symptom burden, and exacerbation rates, aligning with evidence of its potential anti-inflammatory and immunomodulatory effects [16,17]. Although prior RCTs and meta-analyses have shown limited efficacy, such trials were largely conducted in high-income settings with widespread inhaler access. In contrast, our findings highlight that in resource-limited contexts like Pakistan, where inhaled therapies may be costly or inconsistently available, low-dose theophylline could serve as a pragmatic adjunctive option to improve clinical outcomes.

There were marked improvements in spirometric indices. At baseline, the mean FEV₁ was 1.12±0.25 L, which increased to 1.33±0.28 L after 12 months (p<0.001). The mean FVC rose from 2.01±0.42 L to 2.20±0.44 L (p<0.01), while the FEV₁/FVC ratio improved from 55.72±5.11% to 60.45±5.12% (p<0.01). These improvements mirror earlier studies reporting that adjunctive low-dose theophylline enhanced lung function in COPD patients [17,20,21]. Zhou et al. also showed sustained FEV₁ improvement over a year with slow-release theophylline [23]. A meta-analysis [24] of 10 RCTs including 2,771 patients showed that adding oral theophylline to inhaled therapy in stable COPD modestly improved FEV₁ and FVC, reduced exacerbation rates and COPD-related hospitalizations, but did not significantly affect FEV₁% predicted, FEV₁/FVC%, or symptom scores while significantly increasing the risk of drug-related adverse reactions, particularly gastrointestinal events.

Symptom burden decreased significantly. The mean CAT score fell from 22.8±3.6 to 15.6±2.9 (p<0.001), while the mMRC dyspnea grade declined from 2.92±0.61 to 2.02±0.49 (p<0.001). These findings align with prior validation of CAT and mMRC as reliable symptom assessment tools in COPD [18,19] and are consistent with earlier trials where low-dose theophylline reduced dyspnea and improved health status [21-23].

Exacerbation frequency was also favorably impacted. Only 10.61% of patients experienced ≥3 exacerbations in 12 months, while 44.7% reported none. The mean exacerbation rate was 1.12±0.81 per patient (p<0.001). This reduction is comparable to earlier findings from randomized and real-world studies showing that theophylline can lower the frequency and severity of COPD exacerbations [21-23,25].

Safety and adherence outcomes were encouraging. Only 2.27% of patients discontinued due to adverse events, and 88.64% maintained ≥80% adherence. This tolerability profile is consistent with prior reports showing that low-dose theophylline is generally well-tolerated when carefully monitored [15,23]. Moreover, while high-dose methylxanthines are associated with neuropsychiatric and gastrointestinal toxicity [13-15], our findings highlight that careful dosing and monitoring can mitigate these risks in clinical practice.

Mechanistically, our results align with experimental evidence that low-dose theophylline enhances histone deacetylase-2 activity, restoring corticosteroid sensitivity and exerting anti-inflammatory effects [7,20]. Additional work has demonstrated benefits for mucociliary clearance and systemic inflammation reduction [21,23-25]. Regional studies from South Asia and the Middle East also support its functional efficacy and safety in resource-limited populations [16,17], reinforcing the contextual relevance of our findings.

Importantly, while our study does not claim cost-effectiveness in the absence of formal economic analyses, theophylline remains a relatively affordable adjunct in low- and middle-income settings, where inhaler access and affordability remain major barriers [9,11]. Thus, its potential role as a pragmatic adjunct therapy in such contexts warrants further investigation through larger, well-powered trials.

Strengths and limitations

One of the strengths of this research is its prospective design with structured quarterly follow-ups over a complete 12-month period, which enabled the consistent monitoring of spirometric, symptomatic, and clinical outcomes. The use of validated tools such as CAT and mMRC scores strengthened the reliability of symptom assessment, and adherence monitoring enhanced confidence in the treatment effect estimates. Moreover, the inclusion of a relatively large cohort (n=132) from a tertiary care hospital in a low-resource setting enhances the practical relevance of our findings.

Several limitations must be acknowledged. The absence of a randomized control group limits causal inference, and the use of convenience sampling may introduce selection bias. Additionally, the lack of blinding and reliance on self-reported adherence and adverse events may have affected measurement accuracy. The study also did not include an a priori sample size calculation, which limited statistical power for subgroup analyses. Finally, as this was a single-center study, the generalizability of findings to other populations and healthcare systems may be restricted.

## Conclusions

Adding low-dose theophylline to standard COPD treatment over a 12-month period led to marked improvements in clinical outcomes. Lung function improved significantly, with mean FEV₁ rising from 1.12 L to 1.33 L, while symptoms decreased, as reflected by reductions in CAT scores (22.8 to 15.6) and mMRC dyspnea scores (2.92 to 2.02). Exacerbation frequency also declined, with 44.7% of patients experiencing no flare-ups during follow-up. Tolerability was favorable, with over 70% of patients reporting no treatment-related problems, and adherence remained high at 88.6%.

These findings suggest that low-dose theophylline may serve as a clinically beneficial and affordable adjunct to standard COPD therapy, particularly in resource-limited settings where treatment options are restricted. However, as this was an observational study, causal inference cannot be firmly established. Larger multicenter RCTs are warranted to confirm these results and clarify the role of low-dose theophylline within contemporary COPD management strategies.

## Appendices

**Table 6:** Structured questionnaire for data collection COPD: chronic obstructive pulmonary disease; GOLD: Global Initiative for Chronic Obstructive Lung Disease; HTN: hypertension; DM: diabetes mellitus; CVD: cardiovascular disease; CKD: chronic kidney disease; CAT: Chronic Obstructive Pulmonary Disease Assessment Test; mMRC: Modified Medical Research Council; ICS: inhaled corticosteroids; LABA: long-acting beta₂ agonists; LAMA: long-acting muscarinic antagonists

Section	Variable/question	Response options/scale
Consent	Participant information sheet provided	Yes/No
	Participant understood the study purpose	Yes/No
	Voluntary consent given	Yes/No
	Signature/date of the participant	Open-ended
	Signature/date of the investigator	Open-ended
Demographics	Age (in years)	Open-ended
	Gender	Male/Female
	Education level	Illiterate/Primary/Secondary/Higher
	Occupation	Open-ended (e.g., laborer, office worker, retired)
	Socioeconomic status	Low/Middle/High (self-reported)
Smoking history	Current smoking status	Current smoker/Ex-smoker/Never smoker
	Duration of smoking (years)	Open-ended
	Pack-years	Open-ended
Clinical characteristics	Duration of COPD diagnosis (years)	Open-ended
	GOLD stage at enrollment	Stage II/Stage III/Stage IV
	Comorbidities (HTN, DM, CVD, CKD, etc.)	Yes/No (specify if yes)
Spirometry	FEV₁ (L)	Measured value
	FVC (L)	Measured value
	FEV₁/FVC ratio (%)	Measured value
Symptom burden	CAT score	0-40 scale (8 items, 0-5 each)
	mMRC dyspnea scale	0 (none) to 4 (severe)
Exacerbation history	Number of exacerbations in the last 12 months	0, 1, 2, ≥3
	Hospitalization due to COPD in the last 12 months	Yes/No
	Emergency visits in the last 12 months	Yes/No
Treatment and adherence	Current COPD medications	Open-ended (ICS, LABA, LAMA, etc.)
	Theophylline adherence (pill count/self-report)	<50%/50-79%/≥80%
	Patient-reported ease of use	Yes/No
Adverse events	Any side effects reported	Yes/No
	If yes, type of adverse event	Gastrointestinal upset/Headache/Palpitations/Others (specify)
	Severity of the side effect	Mild/Moderate/Severe
	Did the side effect cause discontinuation?	Yes/No
Patient-reported outcomes	Overall satisfaction with therapy	Very satisfied/Satisfied/Neutral/Dissatisfied
	Perceived improvement in symptoms	Much improved/Improved/No change/Worse
